# Male factor infertility and assisted reproductive technologies: indications, minimum access criteria and outcomes

**DOI:** 10.1007/s40618-022-02000-4

**Published:** 2023-01-12

**Authors:** R. Mazzilli, C. Rucci, A. Vaiarelli, D. Cimadomo, F. M. Ubaldi, C. Foresta, A. Ferlin

**Affiliations:** 1 Andrology Unit, Department of Clinical and Molecular Medicine, Sant’Andrea Hospital, Sapienza University of Rome, Via Di Grottarossa, 1036-1039, 00100 Rome, Italy; 2 GeneraLife IVF, Clinica Valle Giulia, Rome, Italy; 3 Department of Surgical Sciences, Gynecologic Unit, University of Rome “TorVergata”, Rome, Italy; 4 Department of Medicine, Unit of Andrology and Reproductive Medicine, University of Padova, Padova, Italy

**Keywords:** Assisted reproductive technology (ART), Severe male factor, Azoospermia, Oligozoospermia, ICSI–IVF

## Abstract

**Background:**

Infertility, which is defined as the inability to conceive after at least 12 months of regular unprotected sexual intercourses, affects about 15–20% of couples worldwide and a male factor is involved in about half of the cases. The development of assisted reproductive technology (ART) made it possible to conceive also to individuals affected from severe oligospermia or azoospermia. However, the impact of the male factor on embryo development, implantation, prevalence of chromosomal abnormalities, genetic and epigenetic alterations, and clinical and obstetric outcomes is still controversial.

**Purpose:**

This narrative review examines the indications, minimum access criteria, and outcomes by individual ART technique in relation to the male factor.

## Introduction

Infertility is defined as the inability to conceive after at least 12 months of regular unprotected sexual intercourse and affects 15–20% of couples worldwide [1]. Male factor per se is responsible for about 30% of infertility cases and an additional 20% as a contributing cause [2]. Semen analysis is the gold standard for the fertility evaluation. Even if it is not able to discriminate between fertile and infertile men, it is extremely useful in monitoring spermatogenesis during and following treatments for male fertility. The latest WHO Laboratory Manual for the Examination and Processing of Human Semen, published in 2021 [3] has introduced some significant novelties in the standardization of human semen processing compared to previous editions. In fact, behind the update of the current methods, the Manual aims to provide an insight in recent developments on semen examination, preparation, and cryopreservation, as well as the quality control and assurance, to better investigate male fertility [3]. Importantly, it highlights that semen analysis alone is not prognostic of fertility, as it is the fertility potential of the couple that defines them as fertile or subfertile, and this represents the most important novelty reported in the Manual. Indeed, semen alteration (before and after treatment) is actually used to decide the therapeutic approach of the infertile couples.

The causes and risk factors for male infertility are numerous and can determine a pre-testicular (hypothalamic or pituitary impairment, with low gonadotropin levels), testicular (characterized by high gonadotropin levels), and post-testicular (with gonadotropin levels generally in the normal range) forms [4]. In addition, risk factors might act pre-puberally or post-puberally, and associated or not with genetic forms (i.e., chromosomal abnormalities and Y chromosomal microdeletion in testicular form and CFTR gene mutation in post-testicular form) [5, 6]. Severe male factor (SMF) includes the conditions of severe oligozoospermia and azoospermia [7].

Treatments exist for many forms of male factor infertility [8]. However, when treatments are not indicated or not effective, Assisted Reproductive Technology (ART) programs can be suggested [8]. This decision also depends on other factors, including the degree to which the couple's fertilizing potential is impaired (maternal age, ovarian reserve, cause of infertility), duration of infertility, and sexual function, and should be done jointly between gynecologists and andrologists [8].

ART are divided into I level, among which the main technique is intrauterine insemination (IUI), and II-III level techniques, namely In Vitro Fertilization—Embryo Transfer (IVF) and Intra-Cytoplasmic Sperm Injection (ICSI) which involve oocyte pick-up and in vitro fertilization [9].

The purpose of this narrative review is to evaluate the state of art on the role of the male factor in the ART, analyzing indications, minimum criteria of access and outcomes for each technique.

## I level

### Indication by male factor and minimum access criteria

Specific indication and accessibility criteria for IUI are mostly fragmentary or absent. Annual reports from the European IVF-Monitoring (EIM) Consortium for European Society of Human Reproduction and Embryology (ESHRE) clearly highlighted different approaches in European countries [10].

National Institute for Health and Care Excellence (NICE) guidelines [9] report difficulty in sexual intercourses (sexual dysfunction or psychosexological issues), sexually transmitted infections (i.e., sperm washing where the man is HIV positive), donor sperm insemination cycles, and same-sex couples as indications for IUI. The Italian guidelines add an important criterion, namely mild-to-moderate male infertility (Table 1). Of note, this last criterion should be implemented only for cases that are not otherwise treatable after relative clinical-diagnostic framing. Idiopathic infertility with normozoospermia is another criterion for accessing IUI.

**Table 1:** Prerequisites, indications and impact on the outcome of the male factor

Technique	Level I	Level II–III	
	IUI	IVF	ICSI
Minimum access criteria	Total IMC with cut-off 1 million	TMC: cut-off 0.2–1 million	–
	Total motility: cut-off 30%	Morphology: cut-off 5% typical forms	
	Morphology: cut-off 5% typical forms		
	TMC: cut-off 5–10 million		
	IMC: cut-off 0.8–5 million/ml		
Indications	Erectile dysfunction or other sexual/ejaculatory dysfunction	Previous IUI failure	As in IVF
	Mild to moderate seminal alteration (not otherwise treatable or failed treatment)	Moderate to severe seminal alteration (not otherwise treatable or failed treatment)	Severe oligozoospermia
	Sexually transmitted diseases (i.e. HIV)		Obstructive and non-obstructive azoospermia (testicular or epidydimal sperm)
	Idiopathic infertility (normozoospermia)		Immotile sperm
			Antisperm antibodies
			Necrozoopermia
			Globozoospermia
Impact on the outcome	As TMC increases, pregnancy rate increases	Reduced fertilization rate, cleavage and blastocyst formation rate associated with SMF	
	No impact from type of semen preparation	Minimal impact of SMF on embryo aneuploidy rate	
	Possible impact of sperm DNA fragmentation on miscarriage rate	Reduction of pregnancy rate by cycle initiated but not by transfer associated with SMF	
	No conclusive data on possible effect of APA	Limited data on neonatal and obstetric outcomes	
		Possible impact of sperm DNA fragmentation on miscarriage rate	
		Possible increased rates of de novo mutations and epigenetic alterations associated with APA	
		Possible increased risk of miscarriage associated with APA	

APA, advanced paternal age; IUI, Intra Uterine Insemination; IVF, In Vitro Fertilization—Embryo Transfer; ICSI, Intra Cytoplasmic Sperm Injection; TMC, Total motile count; IMC, Inseminating motile count.; SMF, Severe male factor

Semen preparation for IUI aims to separate spermatozoa from seminal plasma by replacing it with a culture medium with optimal pH and acid–base balance, so to obtain a high percentage of motile cells and provide a pristine sample. The techniques are simple washing, swim up, and density gradient centrifugation [11]. However, despite the preparation of the semen before the technique, there are minimum criteria for access to the technique. Over time several parameters and cut-offs have been considered, the most frequent are the following (Table 1) [12]:

- Total motility: 30% cut-off.
- Total motile count (TMC), i.e., motile spermatozoa for ejaculate: 5–10 million cut-off.
- These cut-offs are based on studies reporting the motility and TMC as predictors of the pregnancy rate after IUI cycles [12, 13], which highlighted a trend toward an increasing percentage of conception with increasing of these parameters.
- Morphology: 5% normal shapes cut-off. Specifically, Ombelet et al., systematically reviewed sperm parameters cut-offs and found that sperm morphology using strict criteria was the second most cited parameter until 2011, and reported that, in the 68.8% of the 11 studies evaluated, a percentage of normal forms ⩾5 was reported as the best cut-off value to predict IUI outcome [12], even if the 5th Edition of WHO Manual set the cut-off for abnormal forms in 2010 (5th percentile) at 4% [14].
- Inseminating motile count (IMC), i.e., the number of mobile spermatozoa/ml after washing: 0.8–5 millions/ml cut-off. Ombelet et al. [12] found that the specificity of the IMC, defined as the ability to predict failure to become pregnant, was as high as 100%; on the other hand, the sensitivity of the test, defined as the ability to predict pregnancy, was limited. However, total IMC with 1 million cut-off; this parameter was considered a reasonable threshold level above which IUI can be performed with acceptable pregnancy rates.

### Outcomes related with male factor

A recent Cochrane review [11] compared the different preparation techniques and their impact on the clinical outcomes of IUI (clinical pregnancy, current pregnancy rates, multiple pregnancy rates or rates of miscarriage per couple). The authors report insufficient evidence to recommend a specific preparation technique. They also point out that no study assessed the live birth rate. Regarding the impact of seminal parameters on IUI outcomes, most studies report increasing pregnancy rates as TMC increases [15–17]. Specifically, a study based on a large case series reported a pregnancy rate of 12.5% in presence of a TMC > 10 million, which reduced to 5.2% with a TMC < 1 million [15]. Furthermore, a retrospective cohort study based on 2062 IUI cycles highlighted that a TMC < 5 million is associated with a lower pregnancy rate [16]. In partial disagreement, a recent retrospective study of 310 women undergoing 655 IUI cycles showed no live births among the 28 IUI cycles in case of TMC < 2 million [17]. Interestingly, Delaroche et al. reported that the LBR after IUI can be optimized by inseminating a maximum of > 30 million motile spermatozoa [18].

It has been suggested that a high sperm DNA Fragmentation Index (DFI) might impact on fertilizing potential. An extensive study showed that high DFI does not change the pregnancy rate after IUI; however, it is associated with a higher miscarriage rate [19].

Regarding the impact of advanced paternal age and increased Body Mass Index (BMI), the results are controversial. For both, adverse effects on semen quality are known, but specific data on IUI outcome are limited, and no specific age or BMI threshold exists to access the available techniques [20].

## II level

### Indications by male factor and minimum access criteria

The main indications for IVF mainly entail female factor (tubal factor, grade III or IV endometriosis) [9]. Regarding the male factor, the Italian guidelines suggest also moderate-severe grade male infertility, particularly when medical-surgical treatment or previous IUI cycles failed or was considered inappropriate. In case of IVF with cryopreserved semen, there is an indication for II level IVF treatments (depending on the semen quality after warming).

Regarding the minimum access criteria for IVF, the following criteria have been suggested (Table 1) [21]:

- TMC: 0.2–1 million cut-off.
- Morphology: 5–5.5% of abnormal shapes cut-off.

In this regard, recent finding identified a cut-off of 5.5% as a threshold to predict clinical pregnancy after ICSI [22]. These results confirm the potential predictive role of sperm morphology for ICSI outcomes. Furthermore, frozen IVF cycle success appears to be predicted by progressive sperm motility [23]. However, lately the trend in the use of IVF rather than ICSI has been reversed in favor of the latter, also in the presence of male factors suitable for the former. In addition, several laboratories used a "split IVF-ICSI" strategy, which consists in inseminating sibling oocytes with both techniques in similar percentages. Nevertheless, the main indication to ICSI is clear, namely severe male factor (SMF) [19]: obstructive azoospermia (OA) and non-obstructive azoospermia (NOA), after recovery of testicular/ epidydimal spermatozoa; acinesia (i.e., immotile cilia syndrome); round-headed spermatozoa (globozoospermia); anti-spermatozoa antibodies; necrozoospermia (Table 1). Other secondary indications are severe oligozoospermia or cryptozoospermia, especially if after previous ICSI cycles failure with ejaculate sperm or a high rate of sperm DNA fragmentation [24].

In case of azoospermia, the sperm recovery techniques include percutaneous epididymal sperm aspiration (PESA) and microsurgical epididymal sperm aspiration (MESA), where spermatozoa are recovered by needle aspiration or biopsy, respectively, from the epididymis, or directly from the testis by testicular sperm aspiration/fine needle aspiration (TESA/FNA) and the conventional testicular sperm extraction (c-TESE), or micro-TESE (m-TESE).

The Sperm Retrieval Rate (SRR), which is the percentage of cases in which sperm are recovered, is very high in OA (close to 90–100%), while NOA patients, showed an average 50% SRR.

The origin of NOA, the genetic factor, the levels of FSH, inhibin B and the technique used (c-TESE versus m-TESE) were evaluated as potential predictive factors of SRR [25, 26]. A recent meta-analysis of Corona et al. highlighted the importance of testicular volume, founding a value > 12.5 ml as a positive predictive factor [25]. However, to date no predictive factor has been defined; furthermore, since there is no clear evidence of the superiority of any technique, the choice should be done according to patients’ characteristics and ART center experience [8]. Lastly, as for IUI, the choice of additional advanced techniques for sperm selection is based on low-quality evidence [8].

### Outcomes related to male factor

During the early stages of embryo development in vitro, a gap exists between severe oligozoospermic/azoospermic and normospermic patients in terms of oocyte fertilization rate (i.e., number of fertilized oocytes/number of inseminated oocytes) and blastocyst/rate (i.e., number of blastocysts obtained/number of inseminated or fertilized oocytes), testifying the negative impact of SMF [27, 28]. This seems related with a lower maturity, and therefore competence, of the spermatozoa retrieved from the testis [29]. With respect to a putative impact of male factor infertility on embryonic aneuploidies, instead, the data are still controversial. A study by Magli et al. [27] reported a higher prevalence of aneuploidies in NOA patients. However, their analysis was carried out on blastomeres retrieved from cleavage stage embryos in day 3 and analyzed by fluorescent in situ hybridization (FISH). This is an old-fashioned approach, limited to 9 chromosomes (XY chromosomes, 13, 15, 16, 17, 18, 21 and 22) and impairing embryo reproductive competence. More recent data covering 1219 cycles, where chromosomal testing was conducted through 24-chromosome testing techniques on trophectoderm biopsies retrieved at the blastocyst stage, showed an overlapping aneuploidy rate when the cycles were stratified based on the male factor (normozoospermia, moderate oligozoospermia, severe oligozoospermia, OA and NOA) [28]. The sub-analysis conducted within ranges of maternal age and number of oocytes retrieved confirmed this evidence. Therefore, once an embryo reaches the blastocyst stage, the male factor might be less determinant on its chromosomal and reproductive competence. Perhaps the oocytes can "correct" paternally derived genetic errors [30], a hypothesis that deserves deeper investigation.

Regarding the kind of aneuploidies, Coates et al. reported a higher prevalence of sex chromosomes aneuploidies in the embryos produced by patients affected from SMF compared to normozoospermic men [31]. More recently, instead, the prevalence of sex chromosome aneuploidies was evaluated in 7549 blastocysts, and both univariate and multivariate logistic regression analyses showed no association with the male factor [32].

Regarding reproductive outcomes, then, a reduction in the blastocyst rate implies a reduction in the number of transferable blastocysts and, therefore, a reduction in the cumulative pregnancy rate per started cycles but not per single embryo transfer; the percentage of biochemical pregnancies and miscarriage do not appear associated with the seminal profile, except for some reports that claim an impact of sperm DNA fragmentation on higher miscarriage rates [21].

Moreover, obstetrical outcomes also do not appear affected from male factor infertility [21, 28, 33], even when comparing fresh or frozen cycles. Still, long-term follow-up of the newborns including their neurobehavioral development are still necessary.

As for paternal age, although the semen quality appears to worsen in older men, this seems to involve a lower impact on IVF outcomes than maternal age. In this context, Bartolacci et al. suggested that advanced paternal age may impact oocyte fertilization rate and blastocyst rate, yet without interfering with embryo quality, nor with the pregnancy rate [34], while Gallo et al., instead, reported an association between advanced paternal age and lower embryo quality [35]. Some studies suggested a possible impact of advanced paternal age on the risk of miscarriage [36].

Considering the impact of paternal age on embryo chromosomal aberrations, Dviri et al. [37] recently conducted a meta-analysis and found that advanced paternal age is not associated with higher rates of aneuploidy in embryos in an oocyte donation model. On the contrary, a possible increased rate of de novo mutations [38, 39] and epigenetic alterations [40] has been associated with advanced paternal age. More studies are encouraged on this aspect.

## Male factor management before ART

Based on the impact of male factor on IVF outcomes, an adequate diagnostic-therapeutic iter should be guaranteed, aimed at minimizing the actionable features with a well-known impact on fertilization (such as smoking, alcohol, drugs, obesity), along with a proper definition of all other etiopathogenic factors [8, 41].

Medical management includes hormonal and non-hormonal treatment [42]. The main hormonal treatment is represented by gonadotropins [42]. FSH treatment appears effective in men with hypogonadotropic hypogonadism [42, 43], and with oligo/asthenozoospermia with normal FSH plasma levels [8]. Furthermore, it could be useful also to improve non-conventional semen parameters, such as DNA fragmentation. In this regard, Garolla et al. conducted an observational study on 166 infertile men within couples undergoing IVF and reported that FSH hormone treatments reduce sperm DNA fragmentation and increase pregnancy rates [44].

Nevertheless, Santi et al. conducted a multicenter, prospective, observational, clinical practice survey aiming at assessing the therapeutic approaches to the male partners within infertile couples and reported that over 35% of men with idiopathic infertility do not receive any specific treatment before IVF [45]. Among other hormonal treatments, antiestrogens (i.e., clomiphene and tamoxifen citrate) represent possible therapeutic strategies, requiring more evidence [46]. Data, instead, additional hormonal or non-hormonal treatments are either limited or conflicting [47]. Future studies, especially in the context of infections and dysbiosis (antibiotics/anti-inflammatory) [48, 49], autoimmunity (i.e. cortisone treatment) [50], sexual/ejaculatory dysfunctions [51, 52], as well as other endocrine dysfunction (i.e. thyroid or adrenal dysfunctions) [53, 54], are therefore encouraged. In this regard, for instance, Duca et al. showed that in a setting of 320 couples candidates for IVF, whose female partner was younger than 35 years, a "treatable" male factor was noticed in 56% of cases (i.e., OAT with FSH values < 8 mIU/ml, or leukocytospermia/urogenital infections not treated with antibiotic/anti-inflammatory or without eradication, severe varicocele). IVF for them represents overtreatment [55].

## Conclusions

In conclusion, it is critical to define when and which technique to choose for male factor infertility treatment and using IUI as a first-line strategy in case of unexplained and mild/moderate male infertility remains controversial. The male factor appears less determinant than the female one; however, it is desirable to improve spermatogenesis before starting the IVF journey, carefully predicting the true ameliorative possibilities of any treatment and assessing the “window of time available” to this end, by prioritizing maternal age and ovarian reserve as more impacting parameters. Lastly, IVF treatment outcomes can be affected by confounding factors, such as age, reproductive history, duration of infertility, and lifestyle, that should all be carefully evaluated so to properly estimate IVF effectiveness.
